# Medical News and Miscellany

**Published:** 1888-08

**Authors:** 


### Medical News and Miscellany.

PRACTICE AND HOUSE FOR SALE in McLennan county, twelve miles from Waco, at a thriving railroad station and in a rich section of the county. Address O., care Dr. F. E. Daniel, Austin, Texas.

OUR friend and subscriber, Dr. H. H. Darr, of Caldwell, has the JOURNAL'S sincere sympathy in the loss of his bright little son, who died of congestion recently. The little fellow was only six years of age, a promising and unusually intelligent boy, and the pride of his parents. His death is a great grief to them, and a large circle of sympathizing friends.

TRANSACTIONS STATE STATE MEDICAL ASSOCIATION FOR 1888.—The printing of this volume is completed and it is now in the bindery and will be ready for mailing early in September. It is a very handsome volume, of three hundred pages, with an engraved portrait of the President and a cut of the new capitol. Let delinquent members hurry up their dues to Dr. Larendon, the treasurer, at Houston, if they would be remembered by the Publishing Committee.

WE regret exceedingly to chronicle the death of Mrs. T. C. Osborn. She died at Cleburne, Texas, July 18, ult., after a lingering and painful illness, at the age of 61 years. Mrs. Osborn was the wife of Dr. T. C. Osborn (an honorary member of the Texas State Medical Association), and was a native of Alabama, a daughter of Col. Matthew W. McClellan, of Somerville, Morgan county. She was the mother of Dr. J. D. Osborn, of Cleburne. We tender to the bereaved family our sincere sympathy.

EQUIPPING THE TEXAS MEDICAL COLLEGE.—Prof. Hamilton A. West, M. D., of the Texas Medical College Faculty, has gone to New York and Philadelphia to purchase the outfit for the college laboratories and museum, having been selected for that delicate and responsible mission by the Faculty and Board of Trustees. The Professor has full discretionary powers and goes "without instructions as to limit." Everything purchased will be of the latest and most approved style, and the laboratories are to be equipped in the most thorough and complete manner. The regular session will open October 15. Prof. West will contribute a letter to this JOURNAL from New York or Philadelphia.

GOOD OPENING FOR A PHYSICIAN.—The death of Dr. Tompkins, at Sandy Point, near Houston, creates a vacancy in a section of rich agricultural country, which is thus left without a physician within a radius of thirty miles. Mrs. Tompkins is desirous of selling her place, which is the handsomest and most desirable in the town, and will sell it very low. It is modern, newly built, and contains all the conveniences of a first-class home, with orchard, water, flowers, out-houses, etc. Practice worth $3,000 a year, besides the convict practice, for which the State pays $50 a month.

Address, Mrs. F. A. Tompkins, Sandy Point, or the editor of this JOURNAL.

---

DEATH OF DR. FRANK TOMPKINS.—Died July 26, 1888, from the effects of injuries inflicted by an assassin, an account of which was given in the last number of the JOURNAL, Dr. Frank A. Tompkins, of Sandy Point, Texas. Dr. Tompkins was 59 years of age and a native of Edgefield, South Carolina. He came to Texas soon after the war and located at Galveston, where he practiced medicine several years, serving through the memorable epidemic of yellow fever in 1867. He removed thence to the village of Sandy Point, on the Brazos, near Houston, where he resided up to the time of his death, doing a very large and laborious practice, extending over an area of thirty miles. Dr. Tompkins married a Houston lady (name unknown to us) about 1875, and she and five young children survive him and mourn his untimely loss. As a man, a physician, a husband, father, citizen, Dr. Tompkins was most exemplary, and a large circle of devoted friends sincerely mourn his most untimely death, and sympathize with his bereaved family. He was a member of the Texas State Medical Association, and also of the Physicians' Mutual Benefit Association, holding certificate No. 50. (An assessment will be issued for the benefit of his family as soon as the last assessment (No. 7, Dr. Harrington,) is settled up.)

In South Carolina, Dr. Tompkins had an extensive connection amongst the oldest families in the State, the Calhouns and Glovers and others of the old resident families.

---

REWARD.—As an additional inducement to join the PHYSICIANS' MUTUAL BENEFIT ASSOCIATION, this offer is made: Every subscriber who pays his subscription in advance for the present volume (Vol. IV.), and is not a member of the P. M. B. A., will receive his certificate of membership *free*. This offer is good till October 1st. It applies to *new* subscribers as well; but not to those in arrears who pay up, unless they *renew* at the same time, enclosing the subscription due and one year in advance; or, to be better understood, the initiation fee (or dues for year ending December 31, 1888,) is *one dollar;* the subscription price of the JOURNAL two dollars. The two will be clubbed till October 1st for two dollars, the price of the JOURNAL alone.

The annual dues of one dollar is payable yearly thereafter, on January 1st. There is no other expense attending membership, and assessments (of one dollar each) are only made in case of the death of a member, and for the benefit of the widow or beneficiary named in application on joining.

In remitting, state plainly if this offer is claimed; if so, give name of beneficiary, and post-office address.

ANOTHER OFFER.—Any young (or old) physician who desires to aid the cause, is authorized to canvass for membership in the P. M. B. A., and to reserve, as commission, half of the admission fee (or dues for the year ending December 31st), which is one dollar, and also to take subscriptions for the JOURNAL, and retain half the subscription price for his services. Thus, for the two, he can earn $1.50 for each subscriber and member secured, and aid a good cause at the same time.

---

A SPECIAL from Brownsville, of the 14th, says: Quarantine is in force against the neighboring Mexican town of Matamoras, and all communication between the two cities is utterly suspended, to the great inconvenience of hundreds whose business relations embrace both sides of the river, and to the destruction of commerce. The cause for this was, that the Mexican gun-boat, Independencia, which arrived at Bagdad, on the Mexican side of the mouth of the Rio Grande, Friday, two days out from Vera Cruz, landed fifteen passengers and baggage Saturday, and, without any attempt to quarantine them, they came at once to Matamoras, where they arrived yesterday. The Matamoras Junta de Sanidad held a meeting at five o'clock, last evening, with Mayor Milquiades Torres presiding., and, after reading an official communication from the Mexican consul in Brownsville, and the proclamation of our quarantine officer, Wolff, closing the ferry against Matamoras, they took the following extraordinary retaliatory action: "Considering that New Orleans is in the same condition medico-topographically as Vera Cruz, and besides having reliable information that in the first named port there is actually existing an epidemic of diphtheria without this being an obstacle against the admittance of passengers and freight, the Junta, as a measure of prudence and precaution, order that the passes between Brownsville and Brazos and this city, and its jurisdiction, Bagdad, be closed, the time of this prohibition to continue the period that the Junta considers convenient, the Mayor being in accord with the Junta, counting on the official co-operation of the General-in-Chief of this military zone, which has been solicited." This quarantining of cities where there is no infectious disease, is outrageous. Had they taken the most ordinary precaution against the admittance of infection by the passengers of the gun-boat Independencia, or had General Vela chosen to allow his family, who arrived by her, to pass a few days in one of the lower river ranches, all the quarantine difficulty here would have been avoided. As it is, Matamoras has a very small stock of flour, coffee, sugar, and other necessities, in stock, and goods which arrived on the steamer Morgan, this evening, will not be allowed to cross. Matamoras merchants are alarmed, and fear a scarcity, and are treating for a schooner load of groceries to be sent from New Orleans to Bagdad, direct. An attempt was made to send a boat load of citizens of Matamoras to the other side of the river, this afternoon. Among them were several poor women, who were caught on this side, and who are thus separated from their families of little children. The boat was warned off by the Mexican police, and not allowed to land. The river, on the Mexican side, is patrolled by soldiers.

